# Peer Support Experienced by Mothers of Children With Congenital Heart Defects in Sweden

**DOI:** 10.1177/10748407211067788

**Published:** 2022-01-07

**Authors:** Tommy Carlsson, Elisabet Mattsson

**Affiliations:** 1 The Department of Women’s and Children’s Health, Uppsala University, Uppsala, Sweden; 2 The Swedish Red Cross University College, Huddinge, Sweden; 3 Ersta Sköndal Bräcke University College, Stockholm, Sweden

**Keywords:** family caregivers, family/participant group, qualitative, research methodology/design, congenital heart defects, parents, peer support, social support

## Abstract

The aim of this study was to describe experiences of peer support among mothers of children with congenital heart defects. Ten mothers were interviewed through a semi-structured approach and interviews were analyzed with systematic text condensation. The respondents established various channels used for peer support and navigated between the channels depending on what type of information or support they needed. Through the channels, they found peers they developed strong friendships with and who they relied on for emotional support. Communicating with peers involved the reciprocal exchange of unique emotional support between peers who understand each other as well as the exchange of information derived from their collective knowledge, and thus, difficult to find without the help of peers. The findings illustrate the potential strengths of establishing reliable collaboration and liaisons between clinical units and peer support networks.

Congenital heart defects include a wide range of structural birth defects involving the heart and the neighboring blood vessels, with a significant variability in medical severity and prognosis. The incidence of severe congenital heart defects requiring expert medical treatment is between 0.4 and 2.3 per 1,000 live births (Miranović, 2016), and is increasing globally (Liu et al., 2019), illustrating the importance of prenatal and postnatal screening routines (Ewer, 2014; Lytzen et al., 2019). When a diagnosis is established, parents are faced with an unfamiliar and frightening situation resulting in considerable psychological distress, trauma reactions, and impaired quality of life (Bratt et al., 2019; Franich-Ray et al., 2013; Sood et al., 2018). Following the diagnosis, parents are confronted with a complex situation where their own interests and social activities are downgraded in favor of structuring the daily life around their child (Bruce et al., 2014). Some studies suggest that social support decreases the impact a congenital heart defect may have on the psychosocial situation for families and can be a predictor of psychological coping among parents of children with congenital heart defects (Wei et al., 2015). Thus, social support may predict psychological health among parents and is an important factor to consider in pediatric cardiac care. However, the same review concludes a need for more qualitative research that explores the experiences and care needs of these parents.

One way to receive social support is from a person with experience of a similar situation, often referred to as peer support. A defining feature of peer support is that lived experience is utilized when providing emotional support (Watson, 2019). Indeed, empirical research reports that parents of children with congenital heart defects articulate a need for peer support (Bruce et al., 2014), and parents of children with birth defects often establish distance-spanning or face-to-face networks to communicate with peers (Hall et al., 2015). Limited research suggests that peer support between parents of children with congenital heart defects has the potential to result in emotional validation, reassurance, problem-solving, and increased preparedness for future situations (Gramszlo et al., 2020).

While several networks for peer support exist for parents of children with congenital heart defects, few studies have explored the psychosocial effects that these parents experience when communicating with peers. In general, peer support between parents has the potential to offer informational and emotional affirmations that can supplement other types of professional support (Niela-Vilén et al., 2014). More specifically, peer support among parents of children with disabilities can result in shared social identities, mutual exchanges of information, personal growth, and reciprocal benefits related to communicating support to others (Shilling et al., 2013). However, evidence is limited regarding peer support for parents of children with congenital heart defects and more empirical research is needed to draw conclusions about this population. Taken together, information on parents’ perceptions of social support during their child’s disease trajectory could broaden our understanding of how to improve pediatric cardiac care. The understanding of parents’ experiences and needs is imperative for health care providers to be able to formulate and implement appropriate interventions (Wei et al., 2015).

## Aim

The aim of this study was to describe experiences of peer support among mothers of children with congenital heart defects.

## Method

### Study Design

This was a qualitative and exploratory study utilizing an inductive approach. Swedish mothers of children with congenital heart defects were interviewed about their experiences of participating in peer support activities, focusing on experienced benefits and psychosocial consequences related to receiving as well as providing the support.

### Setting

This study was conducted in Sweden, where children with congenital heart defects are provided care at state-run hospitals, primarily via pediatric cardiology units. In addition to medical care, families are offered psychosocial support provided by nurses, social workers/counselors, and psychologists. One national association devoted to children with congenital heart defects and their family members is located in Sweden and offers emotional and economic support to its members (Swedish: *Hjärtebarnsfonden*). The organization arranges regional as well as national peer support activities, including camps where families can meet peers with similar experiences and circumstances. In addition to these activities, parents may also decide to participate in online communities, mainly accessed and managed on the social networking platform Facebook.

### Sample

The respondents were recruited by means of convenience sampling via ads published in two closed Facebook groups for parents of children with congenital heart defects (approximately 900 and 100 members, respectively) and in a newsletter from the national association *Hjärtebarnsfonden*. One mother of a child with a congenital heart defect, who was identified from the clinical network of one of the authors, facilitated access to these closed peer support groups. This parent was a member of both Facebook groups and posted messages in each group containing invitations to participate in the study. Administrators in the national association aided in providing information in newsletters. Initially, respondents participated in an online survey exploring experiences of peer support through written open-ended questions, the results of which are reported elsewhere (*n* = 61 participants in the initial survey; Carlsson et al., 2020). After having submitted their responses in the survey, respondents were asked whether they were interested in participating in this interview study. Eleven mothers and none of the fathers participating in the survey expressed an interest in participating in a follow-up interview. Inclusion criteria were that the respondents needed to be a parent of a living child with a congenital heart defect and that they needed to have experience of participating in either online/distance-spanning or face-to-face peer support activities. When contacted, one mother did not respond to phone calls or emails. Thus, 10 mothers constitute the final sample in this study.

The age of the respondents ranged from 30 to 51 years, with a median age of 40 years. One had two children, whereas the remaining mothers had one child diagnosed with congenital heart defects. All children (*n* = 11) had undergone cardiac surgery. Nine had experience of peer communication via the internet, eight had experience of peer support via the national association, seven had experience of face-to-face peer interactions, and three had participated in peer support via telephone. Table 1 presents sample characteristics.

**Table 1.** Sample Characteristics (*n* = 10 Respondents).

Characteristic	*n*
Age of respondents (years)	
30–39	5
40–49	3
50–59	2
Age of child (years)	
≤3	3
4–12	5
13–18	1
>18	1
Educational level	
High school	2
College or university	8
Time of diagnosis	
Prenatal	2
Postnatal	8

### Data Collection

The interviews were conducted via telephone and with the aid of a semi-structured interview guide (Table 2). The last author, who is a professor, a pediatric nurse, and a midwife with previous experience of performing research interviews, conducted the interviews. At the start of each interview, informed consent was confirmed verbally. The respondents were encouraged to elucidate their responses and follow-up questions were used to promote rich descriptions. All interviews were audio recorded and transcribed verbatim in Swedish. Collectively, the transcribed interviews resulted in 117 pages (1.5 line spacing and 12 font size, Times New Roman) including a total of 50,437 words. The mean interview length was 39 min (median: 36; range: 17–68).

**Table 2.** Semi-Structured Interview Guide.

Main questions	Subquestions
What is your experience of being part of the patient association after your child had been diagnosed with a congenital heart defect?	How did you experience the contact with the association?
	What has the contact with the association meant for you during the pregnancy and after your child was born?
	What has the contact with the association meant for your family?
What is your experience of getting in touch with other parents who have experience of having a child with a congenital heart defect?	In what ways have you had contact with and received support from other parents?
	What has it meant to you to have contact with other parents of children with congenital heart defects?
	How have you experienced the support that the parents have given you during the pregnancy and after the birth of your child?
	When was the support especially valuable?
	Can you please give some examples of positive experiences related to the support?
	Can you please give some examples of negative experiences related to the support?
What experiences do you have of being the one who provides support to parents who find out that their child has a congenital heart defect?	What has it meant for you to provide the support?
	In the future, would you like to provide support to other parents who have recently learned that their child has a congenital heart defect?

### Analysis

The interviews were analyzed with systematic text condensation, an exploratory qualitative method for thematic cross-case analysis. Systematic text condensation is a qualitative descriptive approach, focusing on what the respondents are describing rather than interpreting underlying meanings (Malterud, 2012). The analysis involves steps used to identify and group significant findings in an iterative process. Initially, all transcripts were read in a successive manner, keeping an open mind while reading. After this initial perusal of the transcripts, preliminary themes (*n* = 7) were identified, portraying the general impression of the experiences described by the respondents. The following steps involved identification of meaning units, which were sorted into code groups and subgroups illustrating decontextualized collections of units sharing a similar content. The code groups and subgroups were inspired by the preliminary themes. Condensations were then constructed, defined as artificial quotations written in first-person format and based on the identified code groups. The analyst then rewrote the condensations as reconceptualized descriptions illustrating a cross-case synthetization written in third-person format, and formulated category headings of each code group, portraying the most significant findings within each of the code groups. In total, three category headings were identified during this stage.

The first author performed the analysis, a male specialist nurse, registered midwife, and researcher with previous experience of conducting qualitative analysis including systematic text condensation. The organization of meaning units, code groups, and subgroups was managed with Nvivo for Mac. No automation tools or functions were used in the analysis.

### Ethical Considerations

Respondents provided informed consent to participate after receiving written and oral information about the study. All participants had the option to decline participation at any given time without the need for explanation. Only researchers actively involved in the study had access to the raw data. The study received ethical approval by The Regional Ethical Review Board in Uppsala, Sweden (Approval Number: 2016/366). No monetary or other incentives for participation were provided.

## Findings

### Establishing and Navigating Through Peer Communication by Participating in Channels Serving Different Purposes

The first category heading concerns the methods that the mothers utilized to establish and navigate peer support channels. Overall, peer support was regarded as very important for the mothers, especially among those with a recent diagnosis. However, several experienced difficulties finding peer support networks at the time of the diagnosis and suggested the potential role of health professionals as mediators for establishing peer contacts. By means of word of mouth or through personal invitations, mothers sooner or later found online and face-to-face peer networks. The networks extended over large geographical distances and some respondents had traveled long distances to meet peers face-to-face. On the contrary, some respondents mentioned that peer networks arranged too frequent face-to-face gatherings and that some of these activities were not suited for children with disabilities. Over time, the emotional bond with some peers grew stronger and evolved into close friendships with peers who knew their background and whom they could turn to when feeling an urgent need for support. According to the mothers, the need for peer support varied over time, being high at certain instances, such as when the child was young or in connection with a surgery.

*It’s nice to have someone who you know has followed your journey, so that you don’t always need to establish new contacts with parents when* [staying at the hospital], *when the situation feels the most difficult. . . . Mainly, there are three other parents that I have a really strong relationship with.* (Mother 4, 33 years old)

Mothers described that they used the various peer support channels for different purposes. Communication in smaller and closed online groups was dedicated for peer support concerning intimate topics, whereas larger groups mainly served the purpose of keeping updated on information. In the closed groups, where they had established a mutual trust with other members, respondents felt secure enough to write freely and unconstrained. In other larger groups, with varied members, respondents felt more restricted in their communication. In comparison with the closed Facebook groups, the purpose of the national association and its associated local groups was not seen as a channel for direct communication, but rather, as a means to improve the care and well-being of families and to facilitate peer contacts.

[The national association and the closed peer support groups] *have completely different basic purposes. The* [local association] *was created for, among other things, spreading information about these activities and there is nothing written at all on an individual level, I would say. Such as “now I have this problem with my dear child, can you help me” or “give me tips and advice” or things like that. Nothing of that nature, but rather* [the local association] *only really acts as an advertisement setting for information about activities and the work of the association.* (Mother 7, 45 years old)

Several respondents described an absence of fathers in online communities. Fathers were indeed members in the groups and read messages posted by others, but infrequently participated in discussions with peers. Mothers explained this as being partly caused by many fathers having difficulties sharing their feelings and because of gender-related norms in society. According to the mothers, a separate closed Facebook group was set up specifically for fathers, and they speculated that it was possible that fathers would be more active when mothers could not read their messages. Respondents also discussed that mothers typically take a larger responsibility investigating things related to their situation, and further, that many mothers perhaps experience a greater need to express their emotions than fathers do.

*It has been up for discussion in the group* [that members should] *feel free to add their partners or men or whatever, so that they are also included. And then it’s often—or not often, but it happens—that someone says “yes, but he thinks it is a bit difficult to talk in such large groups*,” *or that many* [fathers] *read but maybe they don’t write. . . . There’s also a subgroup* [for fathers] . . . *I do not really know how big it is, but I know that it has been mentioned on many occasions and I’ve got the feeling that many fathers have turned to it*. (Mother 4, 33 years old)

### Communicating Emotional Support With Peers Who Understand the Unique Challenges Presented to Mothers

The second category heading concerns the unique reciprocal communication of emotional support between peers. One of the main reasons for wanting to communicate with peers was to gain insights on how life is for other mothers in similar situations. Through peer interactions, mothers learned about important aspects of parenthood and could communicate with persons whom they felt were able to understand their situation. In their messages, mothers could tell peers about their own experiences and coping strategies, which provided emotional relief and a sense of comfort. Interacting with peers resulted in feeling less alone, feeling hope, and an increased feeling of togetherness. Communicating with peers felt easier than talking with others, as there was less need to explain basic details. Talking with peers who understand daily life challenges also contributed to feeling acknowledged and less alienated.

*Even if you do not always write or even if you are not always active* [in the peer support groups], *you know that there are people there who actually understand. I think we always need to have a little of that when you end up in a situation, because when you meet people and they say “ I understand” you almost get a little angry because they really don’t understand! But* [in the peer support groups], *you know that the people who are there understand, and just knowing that has been very, very nice.* (Mother 4, 33 years old)

Much of the activities in the peer networks revolved around exchanging emotional support. Mothers described the online community as having an open atmosphere where they could vent their emotional distress in a safe setting. They highly appreciated having an arena where they could write about their thoughts and feelings when being in a particularly emotionally challenging situation. When writing messages, mothers were presented with an opportunity to process their thoughts and emotions. Related to this, mothers described that a potential downside of interacting with peers was that it could bring up difficult emotions and memories. This happened when reading messages from parents of children with very poor prognoses and from expectant parents deliberating over whether or not to continue the pregnancy. Nevertheless, the resounding description was that reading and writing messages from peers mainly involved positive effects on their psychological well-being.

*We* [in the peer support groups] *write things like “now I feel so sad” and “now this has happened” or “now I’ve received this letter” and “now my child will soon have surgery*,” *or whatever it may be, or someone who writes “now my child passed away and now there is nothing left.*” *And then, there are many who write back having been in the same situation. Who may have something to refer to and can write a few lines that “I recognize myself in that” and “thinking of you” or just “giving you a hug” or posting a heart or just something that shows that you kind of understand what it’s all about.* (Mother 9, 39 years old)

Being a member of a closed Facebook group involved a feeling of being able to freely write about their emotions and thoughts without constraints or fear of judgment. Mothers described feeling that they could be honest when interacting with peers and vent their feelings in an allowing arena. Overall, the tone in the closed groups was regarded as nonjudgmental and respectful toward all types of feelings and views, even those that may be regarded as particularly difficult or taboo. The fact that the groups were inaccessible for health professionals made respondents feel comfortable to write more openly about their feelings and experiences.

*Being a closed group for us who are parents, it creates opportunities for a very open climate so that everything can be sort of ventilated, being open about how you think and feel. Also, since the group is closed, no one else* [who is not a member] *can see what is written and said among us when checking their feed. I think that is a big advantage, because you don’t always feel on top and sometimes you just want to vent about what feels difficult, and here* [in the closed peer support group] *you can do that without exposing yourself to too many questions from people who may not be immersed in the world* [we as parents] *live in.* (Mother 4, 33 years old)

### Exchanging Valuable Information Derived From Personal Experiences That Is Otherwise Difficult to Find

The third category heading concerns the exchange of information between peers. By sharing their collective knowledge and communicating advice grounded in their own experiences, mothers could help lessen the impact of stressful challenges presented to peers. From the perspectives of the mothers, parents of children with congenital heart disease become lay experts regarding the daily challenges in their lives. Having collected valuable experiences over the course of several years, mothers could inform and guide one another over the course of parenthood. According to the mothers, parents with recent diagnoses sincerely appreciate hearing and reading honest and uncensored depictions from more experienced peers.

*Last week, someone wrote and was completely shocked. She had been to an ultrasound and had heard the word “wheezing” and she heard the word “ASD”* [Atrial septal defect] *and then she kind of closed down. . . . I wrote that there are many different heart defects and what your daughter has is one of the most common defects and that means this and that, the whistling sound is that faint sound that we talk about . . . she thanked me for the explanation because it was what she needed.* (Mother 3, 50 years old)

Respondents described that the anxiety experienced among many parents with a recent diagnosis was decreased when peers provide them with information and examples derived from their own lives. By communicating about their experiences, peers helped one another prepare for particularly difficult challenges, such as their child’s future surgeries. This included practical information that could be difficult to find on their own, such as when to take parental leave and how to find social benefits available for parents of ill children. Other practical aspects involved school-related topics and how to care for a child with a percutaneous endoscopic gastrostomy.

*You can give concrete examples of things like don’t use up the parental leave without checking if you can get temporary parental benefits, all these practical things, especially because we experienced a lack of such information. It took a very long time before we got to know everything, from temporary parental benefits to care allowance to about schools and preschools and what you can demand, what you can do yourself . . .* [In the support groups], *there are many types of practical advice or where you can turn to get answers to certain questions, and so on.* (Mother 4, 33 years old)

A significant purpose of exchanging information was discussing psychosocial aspects related to having a child with a congenital heart defect, including their own emotional responses as mothers as well as their relationship with the child. Experienced mothers mentioned that they had sufficient knowledge about the medical aspects related to the heart defect, and instead communicate with peers to gain information about other aspects of the complex situation of being a parent of a child with multiple diagnoses, such as neuropsychiatric diagnoses.

*For me*, [other aspects than my child’s heart defect] *have been more interesting, in that he then also has neuropsychiatric difficulties which is what we struggle with as a family right now, much more than his heart defect. We have kind of landed in the heart defect and we know how to deal with it and what has become difficult today is this neuropsychiatric problem . . . The neuropsychiatric part is much bigger for us right now and that’s the type of groups we value most right now.* (Mother 7, 45 years old)

The mothers regarded the main purpose of peer communication as gaining insights into the lives of peers and not as a source of medical information. Nevertheless, peers occasionally helped one another by explaining medical diagnoses, treatment, terms, and other medical facts in a way that felt comprehensible. Mothers mentioned that they appreciated the possibility to write a medical question in the closed Facebook group and quickly receive a response from a peer. This was seen as a way to lessen the burden on health care services, as it led to feeling less urgency to contact professionals when needing an answer to a minor question. Another aspect brought up was the possibility to complement medical information from health professionals with the information from peers, which was particularly appreciated when they had experienced the information provided by professionals as insufficient or confusing.

*We experienced the regular medical doctor as very competent, but he did not always give enough information to the extent we needed, a bit sparse so to say. . . . Often, you want information about the small things. Of course, you also want information about surgeries and other procedures and examinations and things like that. But between parents, there’s much you recognize and you can relate to each other in a different way. . . . I don’t want to burden the health care services with everything and have to ask about the smallest things, if it’s not of a deadly concern. It’s great to have another* [peer] *to discuss with and gain some additional information.* (Mother 5, 37 years old)

## Discussion

In this exploratory study, mothers of children with congenital heart defects were interviewed about their experiences of peer support. The findings illustrate that mothers establish various channels used for peer support and navigate between channels depending on what type of information or support they need. Through the channels, they find mothers who they develop strong friendships with and who they turn to and rely on for emotional support. Communicating with peers involves the reciprocal exchange of unique emotional support between mothers who understand one another. It also involves the exchange of information derived from the collective knowledge and experience of many parents, and thus, is difficult to find without the help of peers.

We explored experiences in a population that has only been scarcely researched in prior studies. From a wider perspective, peer support involves many potential psychological benefits that could help improve the psychological well-being among parents of children with chronic diseases and disabilities (Niela-Vilén et al., 2014; Shilling et al., 2013). The results of our study echo these prior research findings. Research indicates that peer counseling programs have the potential to alleviate mental health illnesses and can result in greater access to psychological support among populations underserved by traditional psychotherapy (Bernecker et al., 2020). According to other studies, subgroups of parents of children with severe and rare illnesses report an unmet need of psychosocial support (Kukkola et al., 2017; Pelentsov et al., 2016). Indeed, mothers interviewed in this study described difficulties finding peer support networks and desired help establishing peer contacts following the diagnosis. Nurses have an important role in this regard, as potential coordinators in family-centered care (Forbes, 2014) who acknowledge the positive effects of peer support and who facilitate contact between parents faced with a recent diagnosis and peer organizations (Bruce & Sundin, 2018). Taken together, the findings call attention to the importance of clinical routines based on collaboration between health professionals and peer networks. In clinical settings, nurses should explore the need for peer support among parents, and when they articulate an interest, be ready to facilitate contact with peer networks.

Previous research has shown that parents of children with congenital heart defects experience a need for additional information that supplements the information provided by professionals. These parents are often presented with unique challenges related to the child’s health, and search for practical information as a means to deal with their activities in daily life (Bellander & Nikolaidou, 2017). According to another qualitative study, parents who have received a prenatal diagnosis of congenital heart defect in the expected child cope with their situation by gathering information (Bratt et al., 2015). In our study, respondents described similar behaviors as they exchanged practical information concerning a wide range of practical and emotional issues with peers, which they highly valued. Moreover, the information received from peers was considered difficult to find on their own, illustrating the unique and impactful potential of peer support within this population. Through the reciprocal exchange of information, online communities involve the process of collective sense-making, in which peers construct meaning by learning from one another (Nakikj, 2019). Our findings illustrate a need for experimental studies investigating the informational benefits of peer support for parents of children with congenital heart defects. Before and after the birth of their child, some parents have a need to hear stories told by other parents with similar experiences. Nurses can ask parents about this and help them gain a deeper understanding and familiarity by relating to others. For example, nurses could collect anonymous depictions shared by their previous patients, which may be shared with parents in need of psychological support. The psychosocial effects of such interventions should be investigated in future studies.

There are methodological limitations that need to be considered when interpreting the findings of this study. This was an exploratory study interviewing 10 mothers, recruited through convenience sampling. The mothers had experiences of different modalities of peer support and represented a range of ages. However, the sample was not varied regarding educational level and gender. All respondents were mothers with higher education and more research exploring experiences among other subgroups is needed. The mothers were recruited by means of convenience sampling, implying that more research is needed that utilizes other recruitment methods. While the chosen recruitment method resulted in all respondents having experience of peer support, we acknowledge that the findings do not illustrate experiences among mothers with negative or stressful experiences of peer support. On the contrary, this study provides novel in-depth findings about a population underserved in research investigating experiences of peer support. Moreover, the respondents were interviewed via telephone, which involves the potential to increase anonymity and promote respondents to feel more comfortable during the interview (Opdenakker, 2006). However, telephone interviews lack the possibility of engaging in and observing nonverbal communication (Sturges & Hanrahan, 2004). While we acknowledge that face-to-face or video interviews could have resulted in more detailed data, our general understanding is that the mothers appreciated the practicality of being interviewed via telephone and we believe that it did not involve any significant loss of data.

## Conclusion

When participating in peer support, mothers of children with congenital heart defects utilize several channels that they turn to depending on their needs. For these individuals, peer support communication involves the reciprocal exchange of both emotional and informational support. Emotional support between peers is highly valued because it is based on a mutual understanding without the need to explain details about the challenges presented to mothers of children with congenital heart defects. Informational support between peers involves collective knowledge, and thus, the opportunity to exchange practical information that is considered challenging to find without the help of peers. Mothers who participate in peer support describe that they, at the time of diagnosis, need more information about peer support networks and want health professionals to initiate contact with peers. Nurses and other health professionals need to carefully evaluate a mother’s need for peer support. Taken together, this study illustrates the potential impact and relevance of peer support interventions in pediatric cardiology care. Our findings imply that nurses should develop, evaluate, and implement peer support interventions for these parents, and that researchers need to systematically assess its potential impact on psychosocial well-being in experimental studies. The findings illustrate the strengths of psychosocial peer support on a personal level for parents in need of psychosocial support. Furthermore, this study highlights the clinical relevance in establishing reliable collaboration and liaisons between health care services and peer support networks.
